# Antibiotic Resistance, Biofilm Genes, and *smeDEF* Efflux Pump in Clinical *Stenotrophomonas maltophilia* Isolates From Iran

**DOI:** 10.1002/mbo3.70222

**Published:** 2026-01-26

**Authors:** Haneen Fadhil Jasim, Nisreen Salah Majeed, Asmaa A. Salam, Rania Hameed Hamad, Yeganeh Behrouzi, Erta Rajabi, Razieh Shahbazi

**Affiliations:** ^1^ Medical Laboratory Techniques Department, College of Health and Medical Techniques University of Al‐maarif Anbar Iraq; ^2^ College of Medicine University of Anbar Ramadi Iraq; ^3^ Biology Department, College of Education University of Fallujah Fallujah Iraq; ^4^ Department of Biology, ET.C. Islamic Azad University Tehran Iran; ^5^ Faculty of Medicine Tehran University of Medical Sciences Tehran Iran; ^6^ Department of Microbiology, School of Medicine Kermanshah University of Medical Sciences Kermanshah Iran

**Keywords:** biofilm, resistance genes, *smeDEF* efflux pump, *Stenotrophomonas maltophilia*, trimethoprim/sulfamethoxazole

## Abstract

*Stenotrophomonas maltophilia* is a nosocomial and opportunistic microorganism with increasing antibiotic resistance rates. This study aimed to assess its biofilm production capacity, antibiotic resistance distribution, and the prevalence of biofilm‐ and resistance‐related genes in clinical isolates. In this multiinstitutional study, 230 isolates were collected from hospitals across Iran between 2022 and 2024. Resistance trends were evaluated using disc diffusion and minimal inhibitory concentration *E* test methods, per Clinical and Laboratory Standards Institute guidelines. Crystal violet staining assessed biofilm production, while polymerase chain reaction (PCR) sequencing identified biofilm‐ and resistance‐related genes. Real‐time PCR was used to evaluate the relative expression of the *smeD*, *smeE*, and *smeT* genes, calibrated against TMP/SMX‐sensitive control strains. Susceptibility rates to trimethoprim/sulfamethoxazole (TMP/SMX), levofloxacin, and minocycline were 97.39%, 93.47%, and 93.04%, respectively. TMP/SMX‐resistant strains showed 19.8‐ and 16‐fold higher expression of *smeD* and *smeE*, compared with sensitive isolates. The *spgM* gene was detected in all isolates, and 93.04% (*n* = 214) were biofilm producers, with most showing moderate‐biofilm formation (*n* = 89, 38.70%). Additionally, the *rpfF* gene was closely associated with strong‐biofilm formation (*p* ≤ 0.05). The *L2*, *L1*, *smqnr*, *sul2*, and *sul1* resistance genes were identified in 214 (93.04%), 181 (78.69%), 135 (58.7%), 136 (59.1%), and 127 (55.2%) isolates, respectively. Our findings demonstrate that most isolates remain sensitive to TMP/SMX, while resistance to alternative antibiotics is rising. Moreover, biofilm production appears significantly associated with the *rpfF* gene.

## Graphical abstract

Iranian clinical *Stenotrophomonas maltophilia* isolates showed high susceptibility to trimethoprim/sulfamethoxazole, minocycline, and levofloxacin, but marked resistance to ceftazidime and ticarcillin‐clavulanate, alongside widespread biofilm production and resistance genes. Overexpressed *smeDEF* efflux pump and *rpfF*‐linked strong biofilms highlight emerging therapeutic challenges.

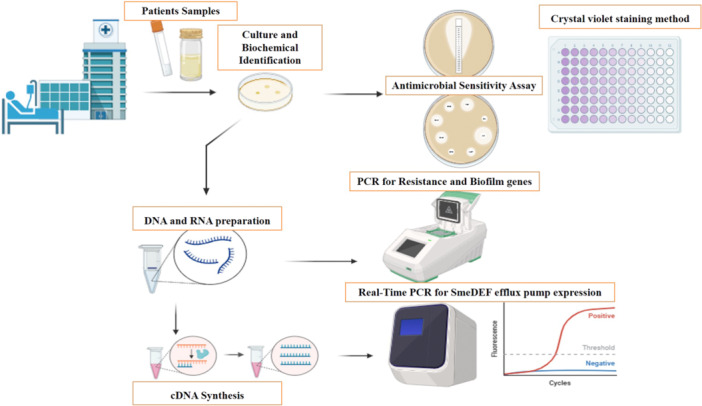

**Abbreviations**
ASA: antimicrobial sensitivity assay
CLSI: Clinical and Laboratory Standards Institute
DHPS: dihydropteroate synthase
LB: Luria–Bertani
LPS: lipopolysaccharide
MDR: multidrug resistance
MICs: minimal inhibitory concentrations
PCR: polymerase chain reaction
*S. maltophilia*
: *Stenotrophomonas maltophilia*
TMP/SMX: trimethoprim/sulfamethoxazole

## 1 Introduction

*Stenotrophomonas maltophilia* (*S. maltophilia*) is a Gram‐negative aerobic microorganism with inherent multidrug resistance (MDR), which is a cause of nosocomial and opportunistic infections, primarily in immunosuppressed individuals, with prevalence rates continuously increasing since 2004 (Quan et al. 2025; AlFonaisan et al. 2024; Cai et al. 2020). Such a pathogen, commonly isolated from the environment, including soil, water, and animals, has been linked to a variety of infections, including bacteremia, mastoiditis, meningitis, endocarditis, and respiratory coinfections with COVID‐19, with mortality rates as high as 77%, owing to biofilm formation on both abiotic surfaces and host tissues (Mikhailovich et al. 2024; Chang et al. 2015; Pompilio, Ranalli, et al. 2020). Many studies regard this pathogen's biofilm production ability as a primary virulence factor, contributing to resistance to the hospital environment and antimicrobial agents, which is believed to develop intrinsically due to low membrane permeability, reduced intracellular drug concentrations as a result of *S. maltophilia*'s efflux pump system activity, the presence of quinolone resistance genes, and rapid resistance gene acquisition via horizontal gene transfer (Brooke 2021; Huedo et al. 2015). All of which, in addition to the delayed progress in developing new antibacterial agents, highlights growing global and public health concerns (Gil‐Gil et al. 2020; Y. Wang et al. 2018; García et al. 2023; Sánchez 2015; Çıkman et al. 2016). Increasing concerns about *S. maltophilia*'s inherent antibiotic resistance have led to the identification of biofilm‐related genes, such as the *spgM* gene, which encodes an enzyme critical to lipopolysaccharide (LPS) production, which is utilized in biofilm formation; the *rmlA* gene, which encodes metabolites required for the exopolysaccharide/LPS‐coupled pathway; and the *rpfF* gene, which regulates key pathogenicity factors and promotes biofilm production (ElBaradei and Yakout 2022; Zhuo et al. 2014; Alavi et al. 2014).

In addition to this pathogen's robust resilience in the environment due to its biofilm formation ability, increasing acquired and inherent antimicrobial resistance in recent years has limited treatment strategies to trimethoprim/sulfamethoxazole (TMP/SMX) and *S. maltophilia*'s high susceptibility rates to TMP/SMX and its pronounced in vitro potency against clinical isolates have resulted in its extensive administration as the first‐line treatment option (AlFonaisan et al. 2024; Emami et al. 2023; Almangour et al. 2024). While global reports indicate that TMP/SMX susceptibility rates remain high, resistance rates have been steadily increasing, with significant regional variability, ranging from 2.35% in Iran to 21% in the United States and Taiwan, and the global mapping of antibiotic resistance trends demonstrates that Africa, Oceania, and Asia have higher TMP/SMX resistance trends than other regions (Mojica et al. 2019; Bostanghadiri et al. 2021; C.‐H. Wang et al. 2017; Bostanghadiri et al. 2024). Furthermore, the *sul2* and *sul1* genes, which encode a variant dihydropteroate synthase (DHPS) enzyme that escapes sulfonamide inhibition, and the *dfrA* gene, which encodes resistant forms of DHPS, are regarded as the primary genes conferring TMP‐SMX resistance (Venkatesan et al. 2023; Ebrahim‐Saraie et al. 2019). *S. maltophilia*'s resistance to β‐lactams, including cephalosporins, carbapenems, and penicillins, is attributed to the *L1* and *L2* genes, and the *smqnr* gene plays a significant role in resistance to quinolones (Brooke 2021; Ebrahim‐Saraie et al. 2019; Huang et al. 2018; Sánchez and Martínez 2010).

Given the increasing resistance trends observed in *S. maltophilia* isolates and the limited data available on the state of resistance patterns and related genes in a middle‐income country like Iran, this study was designed to assess the distribution of resistance‐related genes and the resistance rates to antibacterial agents in clinical isolates.

## 2 Materials and Methods

Between 2022 and 2024, 230 nonduplicate isolates of *S. maltophilia* were retrieved from clinical samples at multiple hospitals in eight provinces of Iran. They were identified via conventional diagnostic biochemical and microbiological methods and subsequently confirmed by polymerase chain reaction (PCR) of the 16S ribosomal RNA gene. Each isolate was stored in 20% glycerol Luria–Bertani (LB) at −70°C until analysis. Quality control strains comprised *S. maltophilia* American Type Culture Collection (ATCC) 13637 and *Escherichia coli* (*E. coli*) ATCC 25922.

### 2.1 Antimicrobial Sensitivity Assay (ASA)

Minocycline (30 μg) and levofloxacin (5 μg) were selected for ASA, which was conducted using the Kirby–Bauer disc diffusion method, per the Clinical and Laboratory Standards Institute (CLSI) guidelines (M100‐Ed35) (CLSI 2025). The minimal inhibitory concentrations (MICs) test strip (Liofilchem; Roseto degli Abruzzi, Italy) was used to determine MICs for ceftazidime, TMP/SMX, and ticarcillin‐clavulanate, with *E. coli* ATCC 35218 and *E. coli* ATCC 25922 strains used for quality control. MIC interpretative criteria followed CLSI M100 35th edition breakpoints (CLSI 2025).

### 2.2 DNA Preparation

Blood agar medium was used to culture the confirmed isolates. Subsequently, the FavorPrep Bacterial DNA Extraction Kit (Favorgen, Taiwan) was employed for DNA extraction, and concentrations and purity were assessed using a Nanodrop (WPA Biowave II Nanospectrophotometer, USA).

### 2.3 PCR Amplification and DNA Sequencing

Using specific primers as illustrated in Table 1, the *smqnr*, S β‐lactamase *L1* and *L2*, *sul1*, and *sul2* genes were confirmed by PCR on a thermal cycler (Eppendorf, Mastercycler Gradient, Germany). This process was performed in a 25 μL reaction containing DNA template (1 μL), primers (10 pmol of each), sterilized distilled water (9.5 μL), and 2× master mix (12.5 μL), including 1× PCR buffer, *Taq* DNA polymerase (0.08 IU), deoxynucleoside triphosphates (0.4 mmol/L), and MgCI_2_ (3 mmol/L). The denaturation step was conducted at 94°C (5 min), with subsequent 36 cycles at 94°C (45 s), primer binding between 50°C and 59°C (45 s), and extension at 72°C (45 s), and the products were then electrophoresed by agarose gel (1%–1.5%), stained with DNA Safe, and visualized with ultraviolet light. PCR amplicons were purified using a PCR purification kit (Bioneer Co., Korea), and confirmation was performed via sequencing analysis, ABI 3730X capillary sequencer (Macrogen, Korea), compared against the NCBI database via the Nucleotide BLAST program (NCBI 2025).

**Table 1:** Sequences of primers utilized.

Primers	Sequences (5′–3′)	Target	References
*16sRNA‐F*	AGTTCGCATCGTTTAGGG	*16s RNA*	Azimi et al. (2020)
*16sRNA‐R*	ACGGCAGCACAGAAGAGC		
*L1‐F*	AGCCGTTAAAATTAAGCCC	*L1*	Azimi et al. (2020)
*L1‐R*	CTTGATTGAAGGGTTGGGCG		
*L2‐F*	CGATTCCTGCAGTTCAGT	*L2*	Azimi et al. (2020)
*L2‐R*	CGGTTACCTCATCCGATC		
*smqnr‐F*	ACACAGAACGGCTGGACTGC	*smqnr*	Azimi et al. (2020)
*smqnr‐R*	TTCAACGACGTGGAGCTGT		
*sul1‐F*	ATGGTGACGGTGTTCGGCATTCTGA	*sul1*	Azimi et al. (2020)
*sul1‐R*	CTAGGCATGATCTAACCCTCGGTC		
*sul2‐F*	GAAGCGCAGCCGCAATTCAT	*sul2*	Azimi et al. (2020)
*sul2‐R*	CCTGTTTCGTCCGACACAG		
*rmlA‐F*	CGGAAAAGCAGAACATCG	*rmlA*	Bostanghadiri et al. (2019)
*rmlA‐R*	GCAACTTGGTTTCAATCACTT		
*spgM‐F*	ATACCGGGGTGCGTTGAC	*spgM*	Bostanghadiri et al. (2019)
*spgM‐R*	CATCTGCATGTGGATCTCGT		
*rpfF‐F*	CACGACAGTACAGGGGACC	*rpfF*	Bostanghadiri et al. (2019)
*rpfF‐R*	GGCAGGAATGCGTTGG		
*smf‐1‐F*	GGAAGGTATGTCCGAGTCCG	*smf‐1*	Sameni et al. (2023)
*smf‐1‐R*	GCGGGTACGGCTACGATCAGTT		
*smeD‐F*	CGGTCAGCATCCTGATGGA	*smeDEF*	García‐León et al. (2014)
*smeD‐R*	ACGCTGACTTCGGAGAACTC		
*smeE‐F*	CGAACTGCATCGGTGATAAC	*smeDEF*	This study
*smeE‐R*	GTTCGCCAATGAGTTCTCCT		
*smeT‐F*	CGG CTTGGTGATTTGGATGT	*smeDEF*	This study
*smeT‐R*	CGTCCAGATCGGTAACGATG		
*rDNA‐F*	TGACACTGAGGCACGAAAGC	*rDNA*	Cho et al. (2012)
*rDNA‐R*	CATCGTTTAGGGCGTGGACTA		

### 2.4 Biofilm Formation

The isolates' ability to produce biofilms was evaluated via the crystal violet staining method, with all experiments run in triplicate (Stepanović et al. 2007). Briefly, overnight cultures were standardized to a 1.0 McFarland standard, diluted (1:100) with tryptic soy broth (200 mL), and then aseptically pipetted into wells of a flat‐bottom polystyrene tissue culture plate (SPL, Korea), which was incubated at 37°C for 24 h. Sterile phosphate‐buffered saline (pH 7.3) was used to wash the wells before fixing the biofilm at 60°C for 1 h. The solution was stained for 10 min with modified crystal violet (250 mL), washed with water, and then air‐dried. Biofilm samples were destained for 20 min using 33% glacial acetic acid (200 µL). The biofilm formation production was measured at 492 nm, based on which biofilms were grouped as (1) nonbiofilm producers (OD < ODc), (2) weak‐biofilm producers (ODc < OD ≤ 2 × ODc), (3) moderate‐biofilm producers (2 × ODc < OD ≤ 4 × ODc), and (4) strong‐biofilm producers (4 × ODc < OD). Additionally, the presence of the *rpfF*, *spgM*, *smf‐1*, and *rmlA* genes was determined using PCR techniques via specific primers detailed in Table 1 (Pompilio et al. 2011).

### 2.5 RNA Preparation and Quantitative Reverse Transcription PCR

The *smeDEF* efflux pump expression in TMP/SMX‐resistant isolates was investigated using specific primers (Table 1) as follows: Suspension cells were prepared and cultured in LB broth and then grown overnight (Cho et al. 2012). The total RNA and DNA extraction processes were performed using the FavorPrep Total RNA Plus Extraction Kit (Favorgen, Taiwan). The Nanodrop (WPA Biowave II Nanospectrophotometer, USA) was used to determine the total RNA concentrations. Complementary DNA (cDNA) synthesis from DNase‐treated RNA was conducted via the SuPrimeScript cDNA Synthesis Kit (Yekta Tajhiz Azma, Iran, Cat. No. YT4500), and the cDNA investigation was performed via real‐time PCR (RT‐PCR). Briefly, each amplification protocol began with an initial denaturation step at 94°C for 10 min, followed by 40 cycles (94°C for 20 s and 59°C for 45 s), with all experiments run in triplicate. The absence of contaminating genomic DNA was confirmed by control reactions omitting reverse transcriptase (‐RT controls). The $2^{‐\Delta \Delta C_{t}}$ method was used to calculate the expression level of the *smeD* gene, following normalization to the rDNA housekeeping gene, and presented as the relative mRNA expression levels compared with TMP/SMX‐sensitive control strains, which were used as the calibrator samples, and *C_t_* was defined as the cycle number at which the exponential increase of the double‐strand DNA (ds‐DNA) first became detectable through fluorescence, generated by SYBR Green I dye binding to the minor groove of ds‐DNA. The expression levels of *smeD* genes were determined using the following formula:

$$n‐\text{fold differences in gene expression}=\frac{\frac{C_{t}\;\mathrm{smeD}\;\text{sample}}{C_{t}\;\text{rDNA sample}}}{\frac{C_{t}\;\mathrm{SmeD}\;\text{calibrator}}{C_{t}\;\text{rDNA calibrator}}}.$$

The *sme* efflux system overexpression was identified at values of *n* > 1.

### 2.6 Ethical Considerations

The present study was approved by the Ethics Committee of Azad University, Tehran, Iran, and was conducted in accordance with the 1964 Declaration of Helsinki. This Ethics Committee served as the central ethics oversight body for the multicenter study, and all participating hospitals across the eight provinces conducted sample collection in accordance with this approval. Written informed consent was obtained from all participants, and patient confidentiality was maintained through coding.

### 2.7 Statistical Analysis

R (version 4.4.2) software was utilized for data analysis, and categorical variables were reported as frequencies. Continuous variables are presented descriptively as means ± standard deviation (SD) without between‐group comparisons, as no a priori hypotheses were tested. The statistical significance of the difference between variables was calculated by Fisher's exact for a 2 × 2 table with any expected count < 5 and the Chi‐square tests for a 2 × 2 table with any expected count ≥ 5, and statistical significance was defined as *p* < 0.05.

The R software (version 4.4.2) was used to generate the figures, including a thematic map displaying isolate counts across provinces, with city‐level polygon data sourced from the geoBoundaries project and downloaded from the Humanitarian Data Exchange platform (https://data.humdata.org/dataset/geoboundaries-admin-boundaries-for-iran-islamic-republic-of). Visualization was performed using the *ggplot2* and *sf* packages.

## 3 Results

### 3.1 Demographic Data and Isolate Information

**Figure 1:**
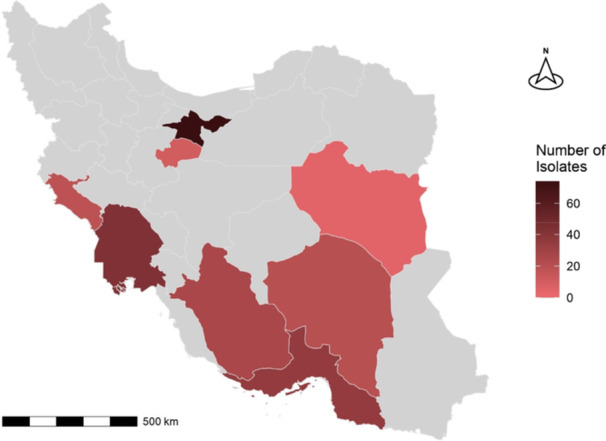
Distribution of isolates across Iran's provinces.

Between 2022 and 2024, 230 isolates were collected from eight provinces across Iran, including Kerman (*n* = 20, 8.7%), Khuzestan (*n* = 43, 18.7%), Ilam (*n* = 18, 7.8%), Hormozgan (*n* = 35, 15.21%), South Khorasan (*n* = 4, 1.7%), Tehran (*n* = 74, 32.17%), Fars (*n* = 26, 11.3%), and Qom (*n* = 10, 4.3%) (Figure 1).

Most samples were obtained from the intensive care unit (*n* = 66, 28.7%), with subsequent collections from the emergency (*n* = 31, 13.5%) and infectious disease units (*n* = 30, 13%). The samples were obtained from 99 females (43%) and 131 males (57%). The study population's age ranged from 4 to 93, with a total mean (± SD) age of 54.09 (± 15.30), and samples predominantly originated from blood (*n* = 170, 73.91%), followed by tracheal tube (*n* = 27, 11.7%), sputum (*n* = 23, 10%), throat (*n* = 9, 3.9%), and central venous catheter (CV line) (*n* = 1, 0.4%) (Table 2).

**Table 2:** Baseline characteristics of the participants.

	*n* (%)
Male	131 (57)
Female	99 (43)
Mean age (± SD^a^)	54.09 (15.30)
Wards	
Postoperative	19 (8.3)
Emergency medicine	31 (13.5)
Immunology	2 (0.9)
Hematology–oncology	4 (1.7)
Gastrointestinal	2 (0.9)
Nephrology	8 (3.5)
ICU^b^	66 (28.7)
Toxicology	3 (1.3)
Infectious diseases	30 (13)
Internal emergency medicine	7 (3)
Surgery emergency medicine	5 (2.2)
Internal	17 (7.4)
General surgery	16 (7)
CPR^c^	8 (3.5)
Cardiology and respiratory	1 (0.4)
Neurology	2 (0.9)
Neurosurgery	2 (0.9)
Urology	6 (2.6)
Infants and neonates	1 (0.4)
Sources	
Blood	170 (73.9)
CV^d^ line	1 (0.4)
Throat	9 (3.9)
Sputum	23 (10)
Tracheal tube	27 (11.7)

^a^ Standard deviation.

^b^ Intensive care unit.

^c^ Cardiopulmonary resuscitation.

^d^ Central venous catheter.

### 3.2 Antimicrobial Resistance Profile

Per CLSI interpretive criteria, levofloxacin and minocycline demonstrated the highest susceptibility rates of 93.47% (*n* = 215) and 93.04% (*n* = 214), respectively, as determined by disk diffusion testing. Table 3 illustrates the MIC ranges, MIC_50_, and MIC_90_, showing a 90.86% (*n* = 209) resistance to ceftazidime, followed by 79.56% (*n* = 183) resistance to ticarcillin‐clavulanate, respectively. Isolates demonstrated the highest susceptibility rates to TMP/SMX (*n* = 224, 97.39%).

**Table 3:** *Stenotrophomonas maltophilia* clinical isolates' antimicrobial susceptibility.

	MIC^a^ (μg/mL)			Disk diffusion number (%)		
Antimicrobial agents	Range	MIC_50_	MIC_90_	Susceptible	Intermediate	Resistant
Levofloxacin	—	—	—	215 (93.47)	—	15 (6.52)
Minocycline	—	—	—	214 (93.04)	—	16 (6.95)
Ceftazidime	≤ 8 to ≥ 32	24	32	—	—	—
TMP/SMX^b^	≤ 2.36 to ≥ 4.76 μg/mL	2	< 2.36	—	—	—
Ticarcillin‐clavulanate	≤ 16.2 to ≥ 128.2	64	128.2	—	—	—

^a^ Minimum inhibitory concentration.

^b^ Trimethoprim/sulfamethoxazole.

### 3.3 Biofilm Phenotypes and Genotypes

**Figure 2:**
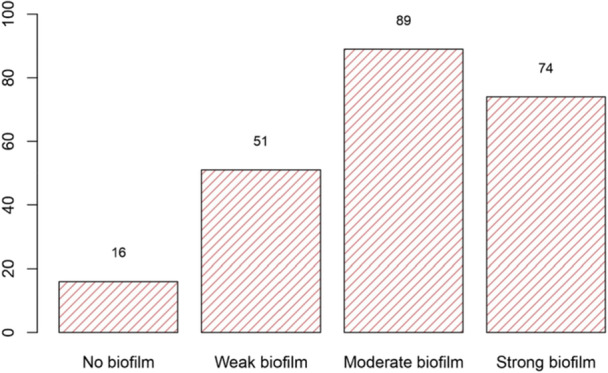
Biofilm formation distribution levels among the *Stenotrophomonas maltophilia* isolates.

Among the total isolates, 16 (6.95%) and 214 (93.04%) were nonbiofilm and biofilm producers, respectively, and of the 214 biofilm‐producing isolates, 51 (22.17%) were weak, 89 (38.70%) were moderate, and 74 (32.17%) were strong producers (Figure 2). The *spgM* gene (*n* = 230, 100%) was the most commonly identified among the biofilm‐related genes through PCR typing, followed by the *smf‐1* (*n* = 214, 93.04%), *rmlA* (*n* = 202, 87.8%), and *rpfF* (*n* = 175, 76.1%) genes. Regarding the nonbiofilm‐producing isolates, the *rmlA* (*n* = 14, 87.5%) and *spgM* genes (*n* = 16, 100%) were the most commonly identified, followed by *Smf‐1* (*n* = 13, 81.3%) and *rpfF* (*n* = 12, 75%). Furthermore, the *spgM* and *Smf‐1* genes were consistently the most commonly observed for biofilm‐producing isolates (Table 4). Moreover, the *rpfF* gene was significantly associated with strong‐biofilm production, respectively (*p* < 0.05) (Table 5).

**Table 4:** Resistance genes distribution across the total isolates.

*L1*	*L2*	*smqnr*	*sul1*	*sul2*	*rmlA*	*spgM*	*rpfF*	*smf‐1*
181 (78.69)	214 (93.04)	135 (58.7)	127 (55.2)	136 (59.1)	202 (87.8)	230 (100)	175 (76.1)	214 (93.04)

**Table 5:** The association of biofilm severity with biofilm‐related genes.

	Biofilm severity							
	No biofilm		Weak		Moderate		Strong	
Biofilm‐related genes	*n* (%)	*p* value	*n* (%)	*p* value	*n* (%)	*p* value	*n* (%)	*p* value
*rmlA*	14 (87.5)	1^a^	41 (80.4)	0.066^b^	80 (89.9)	0.447^b^	67 (90.5)	0.386^b^
*spgM*	16 (100)	—	51 (100)	—	89 (100)	—	74 (100)	—
*rpfF*	12 (75)	1^a^	42 (82.4)	0.234^b^	73 (82)	0.094^b^	48 (64.9)	0.006^b^
*smf‐1*	13 (81.3)	0.088^a^	47 (92.2)	0.759^a^	83 (93.3)	0.919^b^	71 (95.9)	0.280^a^

a Fisher's exact test results.

^b^ Chi‐squared test results.

### 3.4 Prevalence of Resistance Genes

PCR‐based reports on the 230 isolates demonstrated that the *L2* gene (*n* = 214, 93.04%) was the most commonly identified, followed by the *L1* gene (*n* = 181, 78.69%) and the *smqnr* gene (*n* = 135, 58.7%). The *sul1* and *sul2* genes accounted for 55.2% (*n* = 127) and 59.1% (*n* = 136) of the total isolates, respectively (Table 4).

### 3.5 *SmeDEF* Gene Expression Analysis

RT‐PCR was used to investigate the expression of the *smeDEF* gene, considering their TMP/SMX resistance status (MIC ≥ 4/76 μg/mL), which revealed differential expression patterns. In the resistant isolates, the *smeE* and *smeD* genes exhibited 16‐ and 19.8‐fold increased expressions, compared with sensitive strains, respectively, and conversely, the *smeT* gene was downregulated, accounting for a 5.6‐fold decrease in expression (Table 6).

**Table 6:** Differential expression of *smeDEF* genes in resistant isolates compared with sensitive isolates.

Resistance‐related genes	Real‐time PCR *smeDEF*	
	Mean	Change fold
*smeD*	4.3	19.8 ↑
*smeE*	4	16 ↑
*smeT*	−2.5	5.6 ↓

*Note:* The number of TMP/SMX‐resistant isolates = 6, the number of TMP/SMX sensitive isolates = 224, and the fold‐change values are derived from the $2^{‐\Delta \Delta C_{t}}$ method.

Abbreviations: PCR, polymerase chain reaction; TMP/SMX, trimethoprim/sulfamethoxazole.

## 4 Discussion

*S. maltophilia* is a prominent MDR microorganism, which is commonly associated with nosocomial infections and to which increasing trends of resistance to various antibacterial agents have been attributed, subsequently limiting treatment options to TMP/SMX, ticarcillin‐clavulanate, minocycline, tigecycline, colistin, and fluoroquinolones (Chang et al. 2015; Brooke 2014; Sameni et al. 2023; Tamma et al. 2022; Anđelković et al. 2019). This study indicated substantially high susceptibility rates to minocycline, levofloxacin, and TMP/SMX, which are the three mainstay treatment antibiotics for *S. maltophilia* infections (Tamma et al. 2022; Anđelković et al. 2019). The current study demonstrated a 2.6% resistance rate against TMP/SMX in a compilation of *S. maltophilia* clinical isolates, consistent with earlier findings from isolates retrieved from Iranian patients (Bostanghadiri et al. 2021; Sameni et al. 2023; Bostanghadiri et al. 2019). Reports from multiple hospitals in Iran consistently indicate resistance rates of almost 3%, with observed regional variability; studies conducted in the southwestern regions report higher resistance rates (54.1%) compared with those from the northern areas, as geo‐temporal discrepancies and different regional microbial ecologies potentially contribute to the variations in the antimicrobial susceptibility patterns in *S. maltophilia* isolates (Bostanghadiri et al. 2021; Ebrahim‐Saraie et al. 2019; Sameni et al. 2023; Bostanghadiri et al. 2019; Mohagheghzadeh et al. 2020; Dadashi et al. 2023).

The present study also revealed that, except for minocycline and levofloxacin, a significant proportion of isolates exhibited resistance to ticarcillin‐clavulanate (79.56%) and ceftazidime (90.86%). These findings are corroborated by two other multicenter Iranian studies, demonstrating resistance rates higher than the 11% ceftazidime resistance rate reported in a 2020 study (Bostanghadiri et al. 2021, 2019; Mohagheghzadeh et al. 2020). This discrepancy may arise from variations in study designs, since the present study is a multicenter investigation with a sample size that is double that of Mohagheghzade et al.'s study. Moreover, our results are consistent with previous research, indicating increasing resistance rates to ticarcillin‐clavulanate. Taken together, the global resistance rate of 33% against ticarcillin‐clavulanate, with differing regional distributions, suggests its limited efficacy in treating *S. maltophilia* infections (Sameni et al. 2023; Nemati et al. 2015; Banar et al. 2023).

Research from Italy, Brazil, and Egypt indicates that *S. maltophilia* exhibits a strong capacity for biofilm production, with reported rates of 88.2%, 96.7%, and 100%, respectively (ElBaradei and Yakout 2022; Azimi et al. 2020; Pompilio, Savini, et al. 2020). Conversely, our findings indicated that a considerable proportion of *S. maltophilia* isolates exhibited moderate‐biofilm formation capability, and the *rpfF* gene was significantly associated with strong‐biofilm production, which is per previous studies, as moderate biofilm producers consistently outnumber strong‐biofilm‐producing isolates, confirmed by Bostanghadiri et al. who found that weak, moderate, and strong biofilms constituted 28.23%, 37.65%, and 34.12% of their samples, respectively (Bostanghadiri et al. 2021; Sameni et al. 2023; Bostanghadiri et al. 2019; Azimi et al. 2020; Pompilio, Savini, et al. 2020).

The *spgM* is a phosphoglucomutase, contributing to LPS production, which is considered a crucial pathogenicity factor of *S. maltophilia*, and mutations in this gene have been attributed to reduced LPS formation, thereby increasing susceptibility to various antibacterials (Y. Wang et al. 2018). Furthermore, the *rpfF* gene encodes the diffusible signal factor (DSF) responsible enzyme, which participates in the quorum‐sensing of this bacterium, thus regulating biofilm formation and enhancing the resistance of *S. maltophilia*, and comparably, the *smf‐1* encodes the type 1 fimbriae, which facilitate *S. maltophilia*'s adhesion to both abiotic and biotic surfaces and are involved in its biofilm formation process (Mikhailovich et al. 2024; Huedo et al. 2014; Alcaraz et al. 2019). Our analysis of biofilm‐associated genes in clinical *S. maltophilia* isolates indicated that both biofilm‐producing and nonbiofilm‐producing strains primarily contained the *spgM* (100%) and *smf‐1* (93.04%) genes. This finding is supported by existing literature that identifies *spgM* and *rpfF* as the most and least frequently occurring genes, respectively (Bostanghadiri et al. 2021; Sameni et al. 2023; Duan et al. 2020; Divakan et al. 2023). In accordance with our findings, Azimi et al. identified the *spgM* gene in 98% of their isolates. However, they recorded higher and lower frequencies of the *smf‐1* (99.3%) and *rpfF* genes (70%), respectively (Azimi et al. 2020). Furthermore, our results indicated significant associations between strong‐biofilm production and the *rpfF* gene, further confirmed by Azimi et al. who proposed that the biofilm formation ability of *S. maltophilia* may be attributed to the presence of the *rpfF* gene rather than the *spgM* gene (Azimi et al. 2020). The *rpfF* gene encodes a DSF synthase that activates the DSF‐mediated quorum‐sensing system in *S. maltophilia*, regulating virulence‐related characteristics such as biofilm formation and extracellular polysaccharide production, enhancing matrix‐related functions, and fostering a more organized biofilm structure, elucidating the notable correlation between *rpfF* and pronounced biofilm phenotypes identified in this study (Huedo et al. 2015, 2014).

Resistance to penicillins, carbapenems, monobactams, and cephalosporins has been attributed to two distinct β‐lactamases in *S. maltophilia* isolates, including a metallo‐β‐lactamase (*L1*) and an active‐site serine β‐lactamase (*L2*) (Huang et al. 2018; Salahuddin et al. 2018). Moreover, the *sul1*, commonly found in class 1 integrons, and the *sul2*, primarily located on plasmid DNA, encode a DHPS variant that is less susceptible to TMP‐SMX (Toleman et al. 2007; Hu et al. 2011; Barbolla et al. 2004). In addition, topoisomerase IV and gyrase, which are the primary targets of the inhibitory effect of fluoroquinolones, are protected following the encoding of a Qnr‐like protein through the *smqnr* gene expression, allowing fluoroquinolones to bind to this protein (Hooper and Jacoby 2016; Yakout and ElBaradei 2022). In this study, the *L2* (93.04%) and *L1* (78.69%) genes were the most commonly identified resistance‐related genes, with *sul2* (59.1%), *smqnr* (58.7%), and *sul1* (55.2%) following in prevalence, similar to Bostanghadiri et al. who indicated that the *L2* and *L1* genes were the most frequently confirmed in their isolates, both of which are recognized for conferring resistance to β‐lactam antibiotics, thereby increasing resistance to extended‐spectrum cephalosporins (Bostanghadiri et al. 2019; Avison et al. 2001). Conversely, Sameni et al. reported contradictory results to ours regarding the *sul1* and *sul2* genes, noting a higher prevalence of the *sul1* gene, in addition to the higher prevalence of the *smqnr* gene compared with the *sul1* and *sul2* genes (Sameni et al. 2023).

One of the primary MDR efflux pumps in *S. maltophilia* clinical isolates is the *smeDEF* proton pump that increases antibiotic resistance through antibiotic extrusion and comprises *smeD* (the membrane fusion protein), *smeE* (the Resistance‐Nodulation‐Division transporter protein), and *smeF* (the outer membrane channel) proteins, encoded by the *smeD*, *smeE*, and *smeF* genes, respectively. Overexpression in all of which results in increased antibiotics' MICs, and the regulation of the *smeDEF* pump is negatively determined via the *smeT* gene expression (Brooke 2021; Chen et al. 2011; Alonso and Martínez 2000; Wu et al. 2019). In the TMP‐SMX‐resistant isolates, elevated *smeE* and *smeD* expressions were observed, accompanied by a reduction in *smeT* expression, corroborated by the findings of two studies indicating that mutations in *smeT*, a central regulator of the *smeDEF* pump, and deletions in the *smeE* gene result in overexpression and underexpression of the *smeDEF* pump, leading to increased and decreased antibacterial resistance, respectively (Sánchez 2015; Yu et al. 2025). The overexpression of *smeDEF* diminishes the effectiveness of TMP/SMX, constraining treatment alternatives and posing a considerable challenge for patient management due to its role in facilitating resistance to TMP/SMX, the primary treatment for *S. maltophilia* infections (Sánchez and Martínez 2015, 2018). Moreover, *smeT* inactivation, which subsequently results in the *smeDEF* overexpression, has been shown to increase aminoglycoside susceptibility, and the increased concentrations of the *smeF* protein, part of the same complex as *smeD*, are linked to antibacterial resistance, corroborating the results of this study (Sánchez and Martínez 2018; Huang et al. 2017). These results underscore the importance of the *smeDEF* efflux pump as a major factor in TMP/SMX resistance and stress the need for diagnostic methods to detect its overexpression, as well as the development of efflux pump inhibitors or alternative therapeutic strategies to improve clinical outcomes in TMP/SMX‐resistant *S. maltophilia* strains.

Lastly, we would like to acknowledge the limitations of this study, including the predominance of blood samples (73.9%), which may reflect the clinical importance of bloodstream infections and a sampling bias that limits the representation of other infection sites, and differences in sample collection, laboratory methodologies, and resource accessibility among centers, which may affect the results of isolate recovery and ASA. Additionally, the formulation of regional antimicrobial stewardship policies and infection control strategies during the study period may have influenced the reported resistance trends, necessitating cautious interpretation of our findings.

## 5 Conclusion

Given the low resistance rates observed in *S. maltophilia* isolates, our findings support the empiric administration of TMP/SMX as the first‐line treatment option. Additionally, while the relatively low resistance rates to both minocycline and levofloxacin propose such antibiotics as suitable alternative antimicrobials, in cases of resistance to TMP/SMX, our findings raise alarms concerning the increased resistance rates to ticarcillin‐clavulanate and ceftazidime compared with previous research and necessitate urgent interventions, including strengthened infection prevention control measures, to tackle the growing antimicrobial resistance problem in the clinical isolates. Furthermore, regarding the isolates' biofilm formation capability, our study revealed that almost all isolates produced biofilms, with the majority exhibiting moderate‐biofilm production ability. Additionally, a significant association was found between the presence of the *rpfF* gene and strong‐biofilm production.

## Author Contributions

**Haneen Fadhil Jasim:** conceptualization, data curation, investigation, methodology, resources, visualization, and formal analysis, writing – original draft, writing – review and editing. **Nisreen Salah Majeed, Asmaa A. Salam**, and **Rania Hameed Hamad:** conceptualization, data curation, investigation, methodology, writing – original draft, writing – review and editing. **Yeganeh Behrouzi:** project administration, data curation, writing – review and editing. **Erta Rajabi** and **Razieh Shahbazi:** data curation, methodology, project administration, resources, supervision, visualization, writing – review and editing.

## Funding

The authors received no specific funding for this work.

## Ethics Statement

The authors have nothing to report.

## Consent

The authors have nothing to report.

## Conflicts of Interest

The authors declare no conflicts of interest.

## Data Availability

The data sets used and/or analyzed during the current study are available from the corresponding author upon reasonable request.
